# Clinical Results of Transarterial Radioembolization (TARE) with Holmium-166 Microspheres in the Multidisciplinary Oncologic Treatment of Patients with Primary and Secondary Liver Cancer

**DOI:** 10.3390/biomedicines11071831

**Published:** 2023-06-26

**Authors:** Robert Drescher, Alexander Köhler, Philipp Seifert, René Aschenbach, Thomas Ernst, Falk Rauchfuß, Martin Freesmeyer

**Affiliations:** 1 Clinic of Nuclear Medicine, Jena University Hospital, 07747 Jena, Germany; robert.drescher@med.uni-jena.de (R.D.);; 2 Institute of Diagnostic and Interventional Radiology, Jena University Hospital, 07747 Jena, Germany; 3 Department of Hematology and Oncology, Jena University Hospital, 07747 Jena, Germany; 4 Department of General, Visceral and Vascular Surgery, Jena University Hospital, 07747 Jena, Germany

**Keywords:** liver neoplasms, therapeutic embolization, microspheres, holmium-166, radioembolization, TARE, SIRT

## Abstract

Holmium-166 microspheres are used for the transarterial radioembolization (TARE) treatment of primary and secondary liver cancers. In this study, its efficacy regarding local tumor control and integration into the oncological treatment sequence of the first 20 patients treated in our institution were examined. A total of twenty-nine ^166^Ho-TARE procedures were performed to treat hepatocellular carcinoma (HCC, fourteen patients), metastatic colorectal cancer (mCRC, four patients), intrahepatic cholangiocarcinoma (ICC, one patient), and hemangioendothelioma of the liver (HE, one patient). In eight patients, ^166^Ho-TARE was the initial oncologic treatment. In patients with HCC, the median treated-liver progression-free survival (PFS), overall PFS, and overall survival after ^166^Ho-TARE were 10.3, 7.3, and 22.1 months; in patients with mCRC, these were 2.6, 2.9, and 20.6 months, respectively. Survival after ^166^Ho-TARE in the patients with ICC and HE were 5.2 and 0.8 months, respectively. Two patients with HCC were bridged to liver transplantation, and one patient with mCRC was downstaged to curative surgery. In patients with HCC, a median treatment-free interval of 7.3 months was achieved. In line with previous publications, ^166^Ho-TARE was a feasible treatment option in patients with liver tumors, with favorable clinical outcomes in the majority of cases. It was able to achieve treatment-free intervals, served as bridging-to-transplant, and did not prevent subsequent therapies.

## 1. Introduction

Transarterial radioembolization (TARE), or selective internal radiation therapy (SIRT), is used for the treatment of primary malignancies and liver metastases. Microspheres containing beta-emitting nuclides are applied via a microcatheter into the artery supplying the liver tissue containing the tumor(s) [1]. The microspheres flow with the bloodstream and embolize in capillaries, leading to local irradiation. Due to the predominant arterial supply of hepatic tumors by arteries and not by the portal vein, tumor doses are higher than those of non-tumor liver tissue [2].

In an oncologic treatment sequence, the locoregional TARE is indicated when liver tumors, due to their number and size, are unresectable and not amenable to more local treatment (e.g., transarterial chemoembolization, radiofrequency ablation), but there is also no relevant extrahepatic disease that would require systemic treatment. The TARE with ^90^Y-loaded glass and resin microspheres are established treatment options for hepatocellular carcinoma (HCC), intrahepatic cholangiocarcinoma (ICC), and liver metastases of colorectal cancer (mCRC), neuroendocrine tumors (NET), and of other malignancies [3,4,5]. It can be applied in a palliative setting, for downstaging to surgery, and as bridging to liver transplantation.

In 2015, poly-l-lactic acid (PLLA) microspheres loaded with holmium-166 (QuiremSpheres^®^, Terumo, Leuven, Belgium) received Conformité Européenne (CE) approval as a third option for the radioembolization treatment of liver tumors. The main differences between ^166^Ho-containing PLLA microspheres and ^90^Y-containing resin or glass microspheres are the shorter half-life of ^166^Ho (26.8 and 64.1 h), resulting in a higher tissue dose rate after application, and a specific activity of 200–400 Bq per microsphere, which is between resin and glass microspheres, combining a relatively dense tissue coverage with a low embolic effect [6]. Therefore, ^166^Ho-TARE may have advantages for the treatment of certain types of liver tumors, which may depend on their growth rate and radiation sensitivity. The initial studies evaluating the dosage (HEPAR I study), toxicity and efficacy (HEPAR II and HEPAR PLuS studies), and feasibility of its combination with an anti-reflux catheter (SIM study) focused on patients with metastatic colorectal cancers (mCRC) and neuroendocrine tumors (mNET), including up to 37 patients [7,8,9,10]. The HEPAR Primary study then evaluated the safety and efficacy of ^166^Ho-TARE in 31 patients with HCC, showing less than 10% unacceptable toxicity after treatment [11]. 

TARE procedures with ^90^Y-loaded microspheres have been performed in our institution since 2011. In 2019, TARE with ^166^Ho-loaded microspheres (QuiremSpheres^®^, Terumo, Leuven, Belgium) was established as an additional treatment option. This prospective observational study was performed to evaluate our initial experience with ^166^Ho-TARE procedures in the clinical routine of patients with HCC, mCRC, ICC, and hemangioendothelioma of the liver. Its efficacy regarding local tumor control, the variable integration of the method into the oncological treatment sequences, the achievement of treatment-free intervals, and the potential impact on subsequent therapies were analyzed.

## 2. Materials and Methods

### 2.1. Inclusion and Exclusion Criteria

Cases of patients with primary and secondary liver malignancies were discussed within a multidisciplinary tumor board specialized in hepatic-pancreatic-biliary diseases and recommended for TARE therapy. The decision of which type of microspheres to use was made at the discretion of the nuclear medicine specialists and radiologists performing the treatment. All patients who underwent TARE with ^166^Ho-loaded PLLA microspheres were included in the study. Baseline imaging of the liver was carried out with contrast-enhanced, multiphasic CT or MRI, and thoracic-abdominal imaging was conducted with contrast-enhanced CT. A Child-Pugh score of >8, a Karnofski index of <70%, and a tumor load of >70% of the liver were considered exclusion criteria for TARE. The patient’s clinical history and oncologic treatments before ^166^Ho-TARE were retrospectively evaluated. The study was conducted in accordance with the Declaration of Helsinki, and approved by the Ethics Committee of the Jena University Hospital, Germany (Reg. no. 2022–2626).

### 2.2. ^166^Ho-TARE Procedures

**Figure 1:**
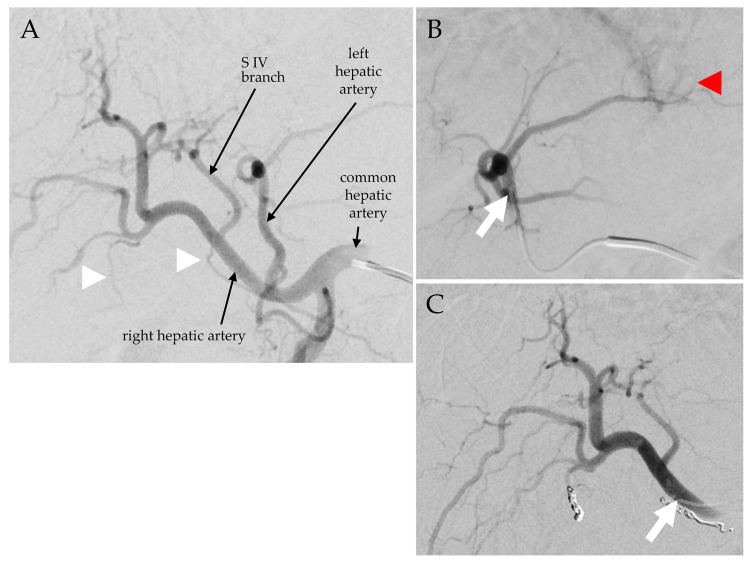
Patient #10, a 77-year-old man with HCC stage II. Pre-therapeutic hepatogram, catheter in the common hepatic artery (**A**). ^166^Ho-TARE of the left liver lobe was performed first, from a catheter position in the left hepatic artery ((**B**), arrow). Some tumor blush is seen corresponding to the lesion in seg. II ((**B**), arrowhead). Before treatment of the right liver lobe, the cystic artery and the aberrant right gastric artery ((**A**), arrowheads) were coil-embolized to prevent extrahepatic microsphere distribution. ^166^Ho-TARE was then performed from a relatively proximal catheter position in the right hepatic artery ((**C**), arrow), including the branch supplying the tumor lesion in segment IVa.

**Figure 2:**
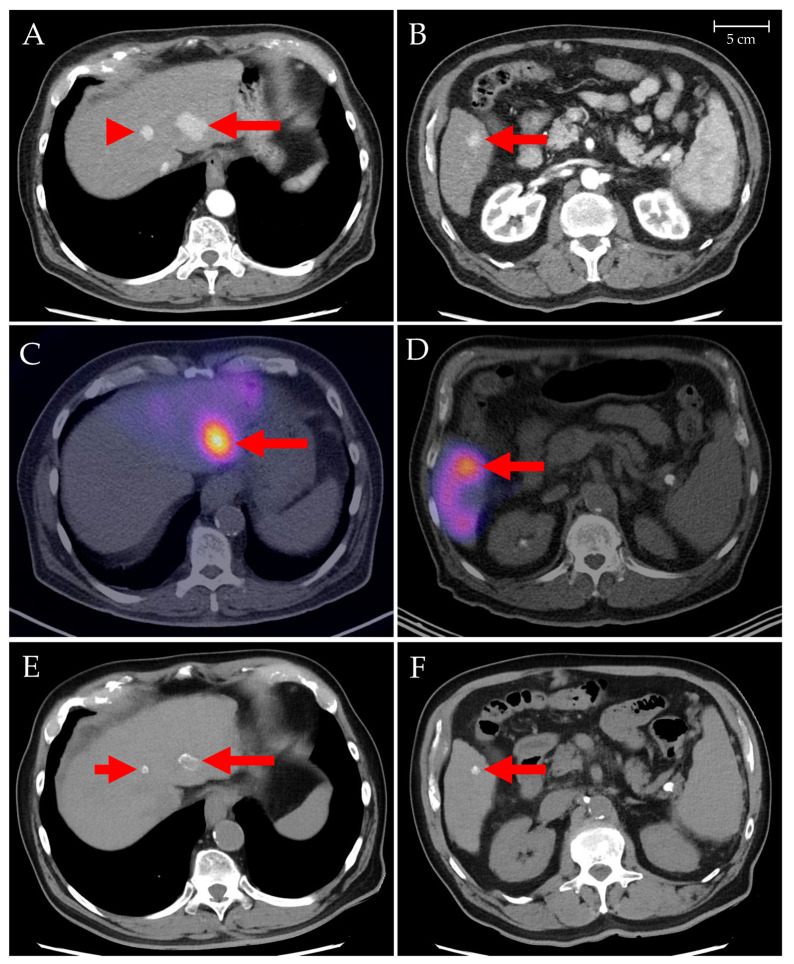
Patient #10. The contrast-enhanced CT showed multiple hypervascularized lesions in both liver lobes, the largest in segments II ((**A**), arrow) and V ((**B**), arrow). The small lesion was in segment IVa ((**A**), arrowhead). SPECT/CT fusion images after left-lobar (**C**) and right-lobar (**D**) ^166^Ho-TARE procedures show distinct microsphere accumulations in the lesions (arrows). Follow-up CT after 3 months revealed complete tumor remission, with lesions decreased in size and without contrast enhancement. Irregular hyperdensities, corresponding to the microsphere accumulations, were noted on non-contrast CT ((**E**,**F**), arrows).

Both TARE planning and treatment procedures consisted of an angiography of the hepatic vasculature, a planar scintigraphy of the thorax and abdomen, and a single-photon emission computed tomography (SPECT) combined with computed tomography (CT) of the abdomen (Figure 1 and Figure 2). For TARE planning, a hepatic angiogram was performed to determine treatment position(s) in the hepatic arterial vasculature supplying the desired liver target area. Arteries distal to this catheter position(s) posing a risk of extrahepatic microsphere deposition were coil-embolized. Then, 150–200 MBq ^99m^Tc-labeled human serum albumin (HSA) B20 microspheres (ROTOP, Dresden, Germany) or 80–170 MBq ^166m^Ho-loaded scout microspheres (QuiremScout^®^, Terumo, Leuven, Belgium) per desired treatment position were slowly injected. On SPECT/CT images, target liver volume (TLV) per treatment position, tracer distribution in tumor and non-tumor tissue, and lung shunt fraction were determined on a syngo.via workstation (Siemens Healthineers, Erlangen, Germany). Extrahepatic tracer depositions were ruled out. The amount of activity (A) required for treatment was calculated using the medical internal radiation dosimetry (MIRD)-based formula A [MBq] = liver dose [Gy] × liver weight [kg] × 63 [MBq/J]. In the HEPAR dose escalation study, a targeted liver absorbed dose of 60 Gy was established, leading to the formula A [MBq] = 3781 [MBq/kg] × liver weight [kg], with a liver density of 1.05 kg/L [9,12,13].

For TARE treatment, the microcatheter was placed in the previously planned position. Setup of the delivery system and injection of the ^166^Ho-loaded microspheres were carried out adhering to manufacturer recommendations [12,14]. Progreat 2.7 F/130 cm microcatheters (Terumo, Leuven, Belgium) were used in all cases. Post-procedural scintigraphy and SPECT/CT were performed on the following day to confirm the distribution of radioactivity, including extrahepatic depositions, which could have led to complications. 

### 2.3. Follow-Up

After the procedures, patients stayed on a nuclear medicine ward for 24 h (TARE planning) or 48 h (TARE treatment). Adverse events occurring during this time and needing medical intervention (e.g., medications) were recorded as peri-procedural. All adverse events were classified according to Common Terminology Criteria for Adverse Events (CTCAE) [15]. Patients were followed-up for a minimum of 24 months after the first ^166^Ho-TARE procedure, or until their death. For initial response assessment, an imaging follow-up with the same imaging modalities as the baseline was scheduled three months after completion of the TARE procedure(s) and served as the basis for further treatment planning. Follow-up was completed 18 months after the treatment of the last patient. 

### 2.4. Outcome Evaluation and Statistics

Overall survival (OS) was calculated as the time from the initial diagnosis of the malignancy (or from the first ^166^Ho-TARE procedure) to the time of death (or to the end of follow-up for patients still alive). Progression-free survival (PFS) was calculated as the time from the first ^166^Ho-TARE procedure to the earliest sign of disease progression on imaging, death, or at the end of follow-up. The treatment-free interval after TARE was calculated as the time between the ^166^Ho-TARE procedure and the earliest of the next locoregional treatment of the same liver lobe, liver transplantation, or systemic therapy. Response to treatment, i.e., complete response (CR), partial response (PR), stable disease (SD), and progressive disease (PD), was evaluated on CT or MR imaging according to the modified Response Evaluation Criteria in Solid Tumors (mRECIST) [16]. Disease control was defined as CR, PR, and SD. Statistical evaluations were performed with SPSS Statistics 26.0 (IBM, Armonk, NY, USA). A *p*-value of <0.05 was considered significant.

## 3. Results

### 3.1. Patient Characteristics and Pre-TARE Treatments

**Figure 3:**
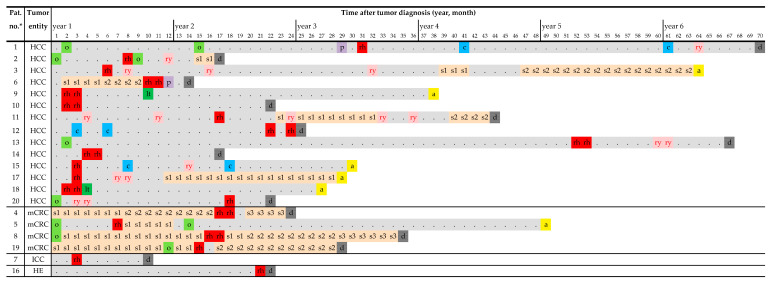
Treatment sequences of patients from cancer diagnosis until the end of follow-up. (Abbreviations: rh, ^166^Ho-TARE; ry; ^90^Y-TARE; o, operation/surgery; s1/s2/s3, first/second/third line systemic therapy; c, chemoembolization; p, percutaneous radiation; lt, liver transplantation; a, alive; d, deceased; *, chronological numbering).

From February 2019 to March 2021, twenty patients (fifteen male, five female; median age 69.5 years, range 57–82 years) were consecutively included in the study. Primary diagnoses were HCC (fourteen patients), mCRC (four patients), ICC (one patient), and hemangioendothelioma of the liver (one patient). Eighteen of the twenty patients (90%) were Barcelona Clinic Liver Cancer (BCLC) stage B (patients #2 and #20: A). A total of 29 ^166^Ho-TARE procedures were performed. The median follow-up interval was 17.7 months (range 0.8–58 months). The clinical characteristics of patients are shown in Table 1, and complete treatment sequences are shown in Figure 3.

In 14 patients with HCC (median age 73.5 years, range 58–82 years), the median interval between tumor diagnosis and ^166^Ho-TARE was 6 months (range 1–51 months). Twelve of the fourteen patients (86%) had underlying chronic liver disease, most commonly alcohol-related liver cirrhosis (Table 1). One patient (#12) had a history of chronic hepatitis C, with negative RNA titers after treatment, and alcohol-related liver cirrhosis. In one patient (#18), the cirrhosis was secondary to hemochromatosis. ^166^Ho-TARE was the first-line treatment in seven patients (50%). Treatments before ^166^Ho-TARE included liver surgery (patients #1, 2, 13, and 20), ^90^Y-TARE (patients #11 and #20), transarterial chemoembolization (TACE; patient #12), percutaneous radiation (patient #1), and systemic therapy (patient #6; first-line sorafenib, second-line cabozantinib). In one patient, a synchronous metastasis was seen in the pelvic bone (patient #6). No further extrahepatic metastases were detected before ^166^Ho-TARE. The Child-Pugh scoring system (CPS) was used to reflect the liver function state in all patients. Thirteen out of fourteen patients (93%) were CPS class A. In 11 patients, the alpha-fetoprotein (AFP) level was below 100 ng/mL. Patients #11, #13, and #14 had AFP levels of 349, 459, and 4677 ng/mL, respectively (normal range <5.8 ng/mL). 

In four patients with mCRC (median age 58 years, range 57–62 years), the median interval between tumor diagnosis and ^166^Ho-TARE was 14.5 months (range 6–16 months). It was not applied as a first-line treatment, but after surgery (patient #5), systemic therapy (patients #4 and #19), or both (patient #8). Before ^166^Ho-TARE, patient #4 received FOLFIRI (folinic acid/fluorouracil/irinotecan)/cetuximab, FOLFIRI/panitumumab, and FOLFOX (folinic acid/fluorouracil/oxaliplatin)/bevacizumab for the treatment of synchronous pulmonary and liver metastases, with the rectal carcinoma (adenocarcinoma, wild-type RAS) in situ. Patient #5 underwent resection of the sigmoid carcinoma (adenocarcinoma, wild-type RAS/BRAF). Liver metastases were detected four months after surgery. Patient #8 underwent resection of the rectal carcinoma (adenocarcinoma, wild-type RAS/BRAF) and FOLFIRI/panitumumab for the treatment of synchronous liver and lymph node metastases, the former being progressive after 14 months. Patient #19 with sigmoid carcinoma and synchronous liver metastases (adenocarcinoma, wild-type RAS) was initially treated with FOLFOX/bevacizumab. The primary tumor was resected 12 months after the initial diagnosis, followed by a right-lobar ^166^Ho-TARE. At the time of ^166^Ho-TARE, the liver function was preserved in all patients (analogous to CPS class A).

Indications other than HCC or mCRC included ICC and hemangioendothelioma of the liver. Patient #7, a 75-year-old man with multifocal ICC, underwent ^166^Ho-TARE as a first-line therapy. Liver surgery was not feasible and systemic options were limited due to multiple comorbidities (including cardiac insufficiency NYHA IV, COPD Gold stage IV, and renal insufficiency stage IV). Patient #16, a 71-year-old woman, was diagnosed with ovarian cancer and treated with an extended hysterectomy followed by carboplatin/paclitaxel. During this systemic therapy, liver lesions suspicious for metastases were detected, but a liver biopsy did not reveal malignant cells. Due to personal reasons, the next follow-up examination was delayed but showed a significant progression of the liver lesions. A biopsy was performed and yielded a hemangioendothelioma of the liver. In retrospect, the tumor growth may have begun almost two years before the histologic confirmation. Given the limited treatment options, the multidisciplinary tumor board recommended bilobar TARE as an individual strategy. 

### 3.2. ^166^Ho-TARE Interventional Procedures

TARE-specific and general outcome data are shown in Table 1. All 29 procedures were performed without technical complications. Procedures were performed with a uni- or bilobar approach (eleven and nine patients, respectively; Table 1). In patients with a bilobar approach, the liver lobe with the higher tumor load (the right lobe in eight patients) was treated first. The other liver lobe was treated after an interval of 6 weeks (median 42 days, range 33–49 days). The lung shunt fraction was below 10%. During five planning procedures and before injecting the planning activity, arteries were occluded to successfully prevent extrahepatic microsphere accumulation (cystic artery: three; cystic and right gastric arteries: one; right gastric artery: one). 

The median prescribed activities per patient and per TARE treatment were 4.7 GBq ^166^Ho (range 1.4–9.1 GBq ^166^Ho) and 3.3 GBq ^166^Ho (range 1.1–5.6 GBq ^166^Ho). For four left-lobar TARE procedures, the prescribed activity had to be divided and injected from two catheter positions (segment IV artery/segment I artery/branch of the left gastric artery, respectively, in addition to the left hepatic artery). The distribution of radioactivity in the liver and tumor tissues after treatment was consistent with that seen in the planning images. No extrahepatic tracer depositions were detected. 

After six procedures, four patients (20%) reported moderate upper abdominal pain starting shortly after the procedures, which responded to oral metamizole and relieved over two days. ^166^Ho-TARE was performed in palliative intent in sixteen patients (%) and as a bridging-to-transplant treatment in four patients (%), respectively. 

### 3.3. Patients with HCC

Three months of follow-up imaging could be performed in 10 of 14 patients. Two patients (#12 and #20) did not undergo imaging follow-up due to fast clinical progression with liver failure/hepatorenal syndrome. In these patients, PD was inferred. Two further patients did not undergo a three-month follow-up due to comorbidities (evolving cardio-renal syndrome, patient #6) and intermittent liver transplantation (patient #18). The resulting disease control rate was 83% (ten of twelve patients): CR in one (8%, Figure 2), PR in seven (58%), SD in two (17%), and PD in two (17%) patients, respectively. In one patient (#15), PD was detected in the untreated liver lobe and treated with TACE. No extrahepatic spread was seen. 

During further follow-up, five patients remained progression-free in the treated liver lobes. In three out of the ten patients with untreated liver tissue, new HCC lesions were detected in the untreated part of the liver before any progression in the treated part (patients #1, #2, #3). Extrahepatic metastases were detected in four patients, abdominal lymph node metastases in #2 and #11, and a singular bone metastasis in #13.

Treatment sequences of the patients after ^166^Ho-TARE were variable (Figure 3). In four patients, it was the final, in two of these, it was the only treatment (patients # 10 and #14). Up to five different treatment options were initiated after ^166^Ho-TARE, including TACE (two patients), ^90^Y-TARE (seven patients; in three cases treatment of residual or recurrent tumor after ^166^Ho-TARE), percutaneous radiation (two patients), and systemic therapy (two patients: sorafenib, two patients: sorafenib followed by cabozantinib). Other than the two liver transplantations, no liver surgery was performed. 

Before ^166^Ho-TARE, 13 out of 14 patients (93%) had a CPS of A5/A6, and one patient of B7. In 8 out of 14 patients (57%), the CPS remained unchanged at the 3-month follow-up. In 4 out of 14 patients (29%), CPS changed from A5 to A6, in three cases due to new ascites. In 2 out of 14 patients (14%) the liver function significantly deteriorated, from CPS A5/B7 to C10, respectively (Table 1). Patient #12 had an HCC stage II, a tumor load in the liver of 3.6%, no known underlying liver disease, and an AFP level of 13 ng/mL. He was initially treated with TACE and underwent sequential ^166^Ho-TARE. Patient #20 also had an HCC stage II, a tumor load in the liver of 3.0%, alcohol-related liver cirrhosis, and an AFP level of 6 ng/mL. Previous liver-directed treatments included bilobar ^90^Y-TARE 14 months before. He underwent ^166^Ho-TARE of the left liver lobe, which represented 30% of the whole liver volume. 

The mean treatment-free interval in the nine patients who received further treatments of the liver tissue targeted by ^166^Ho-TARE was 13.1 months (median 7.3 months; range 2.6–35.8 months) (Table 1). This included patients #9 and #18 who underwent split-liver transplantation while in remission (7.5 and 2.6 months after ^166^Ho-TARE) and were tumor-free 28.1 and 22.5 months after surgery, respectively, and patient #17 with SD who, because of the high tumor load in the liver of 32.1%, underwent ^90^Y-TARE of the same liver lobe after 4.9 months to induce remission. 

**Figure 4:**
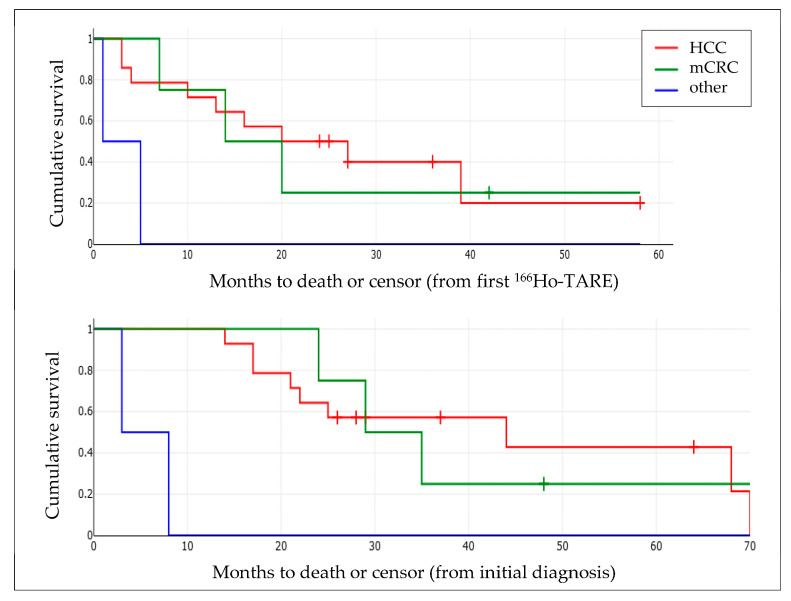
Overall survival of patients with HCC and mCRC after the first ^166^Ho-TARE, and after initial diagnosis of the malignancy.

The mean PFS regarding the treated liver lobe(s) was 14.9 months (Table 2). Mean overall survival after the first ^166^Ho-TARE and after the initial diagnosis of the malignancy was 21.7 ± 15.5 months (median: 22.1, 95% CI: 13.6–29.8 months) and 34.4 ± 19.3 months (median: 27.0, 95% CI: 24.2–44.5 months), respectively (Figure 4).

### 3.4. Patients with mCRC

**Figure 5:**
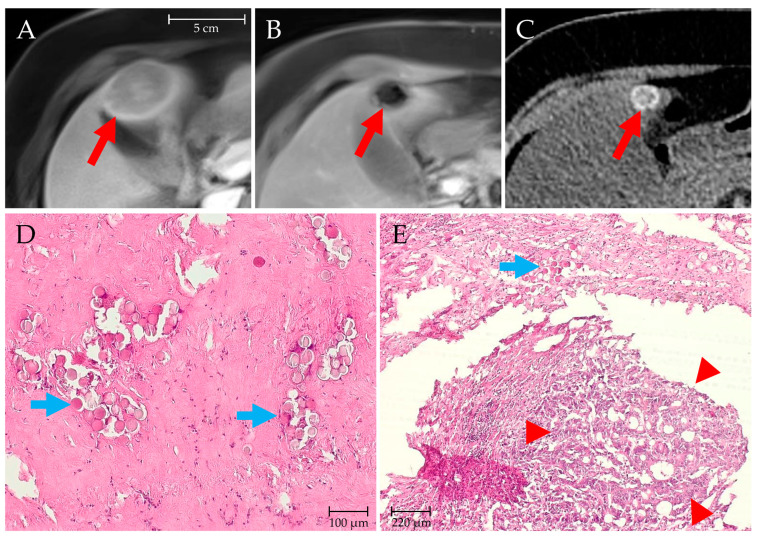
Patient #5, a 62-year-old woman with mCRC. MR showed several metastases in the left liver lobe, the largest in segment IVb ((**A**), arrow, diameter 4.8 cm; T1-weighted sequence, arterial phase). Follow-up MR (**B**) and CT (**C**) imaging three months after ^166^Ho-TARE showed shrinkage of the tumor, with susceptibility artifacts on MR ((**B**), arrow) and focal hyperdensities on CT ((**C**), arrow, diameter 1.5 cm). The patient underwent left hemihepatectomy seven months after the ^166^Ho-TARE. A histopathologic specimen taken from this lesion showed collagenous scar tissue with multiple embedded microspheres ((**D**), arrows, hematoxylin-eosin staining). A specimen from another region of the resected lobe shows a small viable adenocarcinoma lesion ((**E**), arrowheads) and some microspheres in the adjacent tissue ((**E**), arrow).

A variable response to ^166^Ho-TARE was seen in the four patients with mCRC (Table 1). Three months after the procedures, one patient (#8) had CR of liver metastases under continued FOLFIRI/panitumumab treatment. Mediastinal lymph node metastases were detected at 3.2 months, and recurrent liver metastases at 11.8 months after ^166^Ho-TARE. Second- and third-line systemic treatments with FOLFOX/bevacizumab and trifluridine/tipiracil, respectively, were initiated. Patient #5 with left-lobar metastases and no remaining extrahepatic tumor was expected to undergo a living donor liver transplantation in the liver-t(w)o-heal study. The ^166^Ho-TARE was performed as a bridging procedure in combination with FOLFIRI/cetuximab (5 cycles). The follow-up CT revealed nearly complete remission of the metastases, and a left hemihepatectomy was performed. Histology showed predominant scar tissue in the regions of the metastases, minor residual tumor tissue, and microsphere remnants (Figure 5). No further treatment was necessary until the end of the follow-up 41 months later. 

Two patients had progressive disease at a 3-month follow-up. Patient #4 with bilobar progression of the liver metastases and new lung and bone metastases subsequently received a third-line systemic therapy with trifluridine/tipiracil. In patient #19, progressive liver metastases were detected by sonography 1.5 months after ^166^Ho-TARE in both treated and untreated liver lobes despite parallel FOLFOX/bevacizumab. Systemic treatment with FOLFIRI/cetuximab was initiated, but multiple lymph nodes and lung metastases occurred 12 months later. The liver function remained stable (three out of four patients) or deteriorated slightly (one patient, #19) after ^166^Ho-TARE procedures. Two patients developed ascites. 

In patients with mCRC, the ^166^Ho-TARE did not result in relevant liver-treatment-free intervals (mean 0.9 months, median 1.1 months; range 0–1.5 months) (Table 1). In three patients, the ^166^Ho-TARE was carried out under ongoing systemic therapy (patient #8) or the continuation of such therapy had already been planned (patients #5 and #19). Only in patient #5 was the introduction of a third-line systemic therapy a new decision given intrahepatic PD and new extrahepatic metastasis. 

The mean PFS regarding the treated liver lobe(s) was 5.3 months, excluding the patient who underwent resection of the treated lobe (Table 2). The mean overall survival after the first ^166^Ho-TARE and after the initial diagnosis of the malignancy was 20.6 ± 15.1 months (median: 16.7, 95% CI: 12.7–28.5 months) and 34.1 ± 10.6 months (median: 32.2, 95% CI: 21.4–46.9 months), respectively (Figure 5). 

### 3.5. Patients with ICC or Hemangioendothelioma of the Liver

Patient #7 with an ICC underwent ^166^Ho-TARE of the left liver lobe, resulting in a partial tumor response after 3 months (Table 1). The liver function moderately deteriorated, and ascites developed. No further treatment was initiated until the patient’s death 5 months later. Patient #16 with a hepatic hemangioendothelioma had a tumor load of 58% of the liver, predominantly involving the right liver lobe. Of the planned bilobar treatment, only a ^166^Ho-TARE of the right liver lobe was performed. Shortly afterward, the liver function deteriorated. Ultrasound showed disease progression in both liver lobes. The patient died due to hepatorenal syndrome. 

## 4. Discussion

PLLA microspheres loaded with ^166^Ho represent the third type of microspheres to be used for the TARE of liver tumors. The clinical indications are similar to those for ^90^Y-loaded resin and glass microspheres, but the ^166^Ho-PLLA-microspheres may, due to their different physical characteristics, have advantages in certain types of tumors. TARE treatments have been shown to be effective as a first-line treatment, particularly in patients with HCC, and also in the salvage situation [1]. It is also known that a multimodal and multidisciplinary approach yields the best oncologic results [17]. Currently, concluded and ongoing ^166^Ho-TARE studies aim to establish the safety and efficacy of the procedure for indications already evaluated for ^90^Y-TARE, and to gain experience in clinical practice. In our institution, ^166^Ho-TARE has recently been added to the treatment portfolio for liver tumors. Until now, it has been mainly used for unresectable HCC to delay the initiation of systemic therapy, and in some patients with mCRC (Figure 2).

### 4.1. Procedural Characteristics and Technique

The ^166^Ho-TARE treatment sequence is similar to those with ^90^Y-containing microspheres. A planning procedure is performed to evaluate the vascular anatomy of the liver and, by injecting a surrogate compound at selected catheter positions, to determine the distribution of microspheres in the liver, acknowledge extrahepatic depositions, and calculate the lung shunt fraction. The planning procedure can be conducted with ^99m^Tc-labeled humane serum albumin (HSA B20; ROTOP, Fürstenfeldbruck, Germany) and ^99m^Tc-labeled macroaggregated albumin (MAA). Another option is a dedicated scout dose (QuiremScout^®^; Terumo, Leuven, Belgium), which contains the same microsphere type as that for treatment: PLLA microspheres of the same size and density, but loaded with a low activity of ^166^Ho (max. 250 MBq). The ^166^Ho scout dose has been shown to be safe and to predict microsphere distribution better than ^99m^Tc-MAA [18,19]. The application system allows the alternating application of microspheres and flushing with saline and/or contrast media. To minimize residual activity in the application system, additional flushing steps should be performed when treating with prescribed activities below 1.0 GBq of ^166^Ho [20]. We did not experience technical difficulties during the microsphere application. In some patients, a temporary mild slowing of the blood flow in the hepatic arteries was observed, but no stasis occurred. Peri-procedural complications were less frequent than in other studies (up to 20% of patients) and presented as upper abdominal pain, consistent with a mild post-embolization syndrome [8,11].

### 4.2. Clinical Outcomes

In the HCC group, a disease control rate of 83% at three months and a median PFS of 10.3 months was achieved in the liver tissue treated with ^166^Ho-TARE (Table 2). The median OS of these patients was 22.1 months. The two studies evaluating ^166^Ho-TARE concluded until now reported a disease control rate of 89% (at six months, nine patients included) [21] and complete or partial response in 54%/84% (at three/six months, thirty-one patients included) [11]. In the first study, no death occurred at six months of follow-up. In the second study, the median OS was 14.9 months.

Radioembolization is an established bridging therapy to delay hepatic tumor progression and to ensure that patients survive the waiting time for a liver transplant without developing contraindications for transplantation [22,23]. Since the waiting times for liver transplants are steadily increasing, an adequate bridging therapy can be crucial for the individual patient. There are several approaches for bridging in patients with HCC: transarterial approaches (chemoembolization, radioembolization), local-ablative measures (microwave or radiofrequency ablation), percutaneous radiation, or even systemic therapies (e.g., sorafenib and lenvatinib). The choice of therapy depends on the current national guidelines, the expertise and preference of the individual centers, as well as on the tumor characteristics (the size and number of tumor nodules and localization). In two HCC patients of our cohort, split-liver living donor transplantations were performed without complications. Both patients remained tumor-free. 

The largest database for the ^166^Ho-TARE exists for patients with mCRC. Phase I and II studies confirming their safety and efficacy have mainly evaluated patients with this type of metastases, and a further study including a comparison of a standard and an anti-reflux catheter has been added (HEPAR I/II and SIM studies [8,9,10]). The median OS intervals were 13.4 and 7.8 months (HEPAR II/SIM, respectively), applying the 60 Gy average dose to the target liver. In the HEPAR II study, the median time to progression was 3.0 months [8]. The median overall survival after ^166^Ho-TARE of the four mCRC patients in this study was 16.7 months. Contrary to HCC patients, the median PFS in the untreated liver was longer than in the treated liver (21.7 and 2.6 months, respectively; Table 2). A patient with large left lobe metastases could undergo a left hemihepatectomy after the combination of ^166^Ho-TARE with FOLFIRI/cetuximab (patient #5). She remained tumor-free. 

In this study, only one patient with an unresectable ICC underwent ^166^Ho-TARE. Locoregional treatments including TARE are not recommended as a first-line option for this type of tumor by international guidelines. In this case, it was deemed appropriate because of limited alternatives [24]. ^166^Ho-TARE induced PR and treated-liver PFS was longer than untreated-liver and extrahepatic PFS. A combination with cisplatin/gemcitabine chemotherapy was not possible due to the patient’s comorbidities [25].

### 4.3. Adverse Events during Follow-Up

In the majority of patients (55%), liver function, represented by the CPS, remained stable after ^166^Ho-TARE (Table 1). In six patients (30%), the CPS increased by 1, which was caused by new ascites in five cases, not by laboratory changes. Symptoms of ascites often lead to a worse quality of life for the patients (abdominal swelling, weight gain, less agility). In the HCC group, the liver function of two patients significantly deteriorated shortly after treatment (patients #12 and #20). In patient #12, the cause may have been the underlying liver disease of alcohol-related cirrhosis, a history of Hepatitis C, and the ^166^Ho-TARE of both liver lobes. However, the pre-procedural CPS was A5, and the treatment was considered possible. Patient #20 already had a pre-procedural CPS of B7 with mild ascites. The target volume of the ^166^Ho-TARE was only 30% of the whole liver volume, but the patient had alcohol-related cirrhosis and a history of a bilobar ^90^Y-TARE. Due to the clinical situation, neither patient underwent further liver imaging, and a concurrent PD could not be ruled out. 

The third patient with fast liver function deterioration was the woman with hemangioendothelioma of the liver (patient #16, tumor load 58%). The treatment was indicated because of limited alternatives and a favorable result described in the literature, where a patient with the same diagnosis was successfully treated with ^90^Y-TARE [26]. We decided to use ^166^Ho microspheres, because of the shorter half-life and potentially faster onset of action. The patient died less than one month after the first of two planned ^166^Ho-TARE. Because of the extensive tumor load of 58% and preexisting liver cirrhosis, the TARE may have accelerated the liver failure. Best supportive care should have been prioritized.

### 4.4. Achievement of Treatment-Free Intervals

An advantage of the locoregional treatment of liver cancers is the achievement of treatment-free intervals, avoiding the adverse effects of systemic therapy in particular, which is very important for the patient’s quality of life. In the patients with HCC who underwent further treatments after ^166^Ho-TARE, treatment-free intervals of up to 35.8 months (mean 13.1 months) could be achieved. In patients with mCRC, the ^166^Ho-TARE did not achieve prolonged treatment-free intervals (Figure 3). It was performed during systemic therapy, or such therapy was initiated/changed shortly thereafter, in two patients to treat extrahepatic metastases (patients #4 and #8). 

### 4.5. Standard and Personalized Dosimetry

In our patient cohort, the prescribed activity was calculated with the MIRD formula, solely based on the target liver volume, to reach an average dose in this volume of 60 Gy. This radiation dose was determined as the maximum tolerated radiation dose (MTRD) in the HEPAR I dose-escalation study [9]. Individualized treatment plans may improve treatment outcomes. The SPECT/CT data of the planning procedure are then analyzed by a dedicated dosimetry software (e.g., Q-Suite^®^ v 2.1, Quirem Medical, Deventer, The Netherlands or MIM^®^, MIM Software, Cleveland, OH, USA), which allows a voxel-based determination of the activity distribution in liver and lung and a prediction of tissue doses after ^166^Ho-TARE. By optimal balancing tumor and non-tumor radiation doses, clinical outcomes may be improved while minimizing liver function impairment. A dose-response relationship has already been established for the treatment of CRC metastases [27]. A dose-finding study for early-stage is ongoing [28].

Currently, according to the manufacturer’s recommendations, a dose escalation can only be considered to exceed the recommended average liver dose of 60 Gy when part of the liver is treated [29]. In our study, the mean PFS in the untreated liver tissue was shorter (10.9 months) than that in the treated tissue (Table 2). A progression in untreated liver tissue means that new lesions have occurred there, and a TARE may be necessary that was not initially planned. The permanent risk of new HCC lesions in previously tumor-free liver segments and the liver functional reserve must be kept in mind during dose planning, since further potentially liver-function-impairing treatments may be necessary. 

### 4.6. Limitations of the Study

The main limitation of this monocentric, non-randomized observational study is the small number of patients with different tumor types and oncological treatments. ^166^Ho-TARE was performed in different tumor stages, and the treatment sequences were highly variable, which makes an evaluation of its effect beyond the liver target lesions and of the impact of other treatment modalities difficult. Evaluation after ^166^Ho-TARE was focused on technical success, tumor progression, and OS. Therefore, liver functional changes and clinical adverse events may have been underestimated. 

In our study, the Child-Pugh scoring system was used to estimate the liver function state and to detect changes after TARE. Other tools to model the functional liver reserve before and after locoregional treatments, such as the albumin-bilirubin (ALBI) or the platelet-albumin-bilirubin (PALBI) score, should be considered in future research for response prediction and assessment [30].

Not all patients in this study underwent MR imaging of the liver in addition to CT. Despite multiphasic CT being a recognized modality for oncologic liver imaging, MRI has a higher sensitivity and specificity, particularly for the detection of early-stage HCC [31]. 

## 5. Conclusions

This study aimed to reflect the clinical complexity and variable oncologic settings when indicating ^166^Ho-TARE treatments and to evaluate its advantages and disadvantages in relation to other therapies a patient may undergo. In this real-world patient cohort, ^166^Ho-TARE proved to be a feasible treatment option in patients with liver tumors, with good to very good clinical outcomes in the majority of cases. The therapy was able to achieve treatment-free intervals, served as bridging-to-transplant, and did not prevent subsequent therapies. Combination with systemic therapies in patients with mCRC was feasible; in this setting, the ^166^Ho-TARE can be seen as supporting therapy. However, the small study size, containing different tumor types and oncologic treatments, needs to be considered when drawing treatment recommendations. Diverse treatment sequences, with the TARE as the first, the final, or an interim treatment in between other locoregional and systemic therapies, indicate that the best sequence has yet to be found. The exact value of ^166^Ho-TARE in different tumor entities, as distinct from ^90^Y-TARE, and the potential advantages of personalized dosimetry planning have to be determined.

## Figures and Tables

**Table 1:** Patient characteristics, treatment data, and survival.

Patient and Disease Characteristics				Status prior to ^166^Ho-TARE				^166^Ho-TARE Procedures			Status at 3 Months Follow-Up		Progression-Free Survival after ^166^Ho-TARE			Treatment-Free Interval	Overall Survival		Cause of Death
Patient no.*	Age (yrs.), Gender	Tumor Entity	Underlying Liver Disease	Tumor Stage	Tumor Volume (mL)	Tumor Load (%)	Liver Function State (CPS)	Proportion of the Liver (%)	Prescribed Activity (GBq)	Periprocedural Adverse Events	Liver Function State (CPS)	Treatment Response	Treated Liver (mos.)	Untreated Liver (mos.)	Extrahepatic (mos.)	(mos.)	After ^166^Ho-TARE (mos.)	After Initial Diagnosis (mos.)	
1	73, m	HCC	cirrhosis	II	24	7	A5	27	1.4	none	A5	PR	34.1	9.0	38.7	35.8	38.7	69.6	LF
2	75, m	HCC	NAFLD	IVB	19	4	A6	29	1.8	none	A6	PR	6.6	3.6	6.6	7.0	9.5	17.1	prM
3	66, m	HCC	none	IIIA	87	6	A5	56	5.6	none	A5	PR	32.5	8.6	58.0	32.7	58.0	63.5	-
6	81, m	HCC	cirrhosis	IVB	288	14	A5	100	3.3, 5.0	none	A6	n.a.	4.4	-	4.4	n.a.	4.4	14.1	CRS
9	58, m	HCC	cirrhosis	II	67	5	A5	68	2.5, 2.9	none	A5	PR	LTx	LTx	35.6	7.5	35.6	37.1	-
10	77, m	HCC	cirrhosis	II	17	2	A5	63	1.1, 2.2	none	A5	CR	19.9	19.9	19.9	n.a.	19.9	21.7	LF
11	65, m	HCC	cirrhosis	IIIA	223	28	A5	21	3.2	none	A6 ^A^	SD	5.6	14.6	22.2	6.2	26.5	43.7	-
12	60, f	HCC	cirr., Hep. C	II	40	4	A5	100	2.6, 1.8	none	C10 ^A^	PD*	3.0	-	3.0	n.a.	3.0	25.0	LF/HRS
13	81, m	HCC	none	IIIA	365	22	A5	100	5.5, 1.1	abd. pain	A5	PR	7.2	-	15.8	8.4	15.8	67.8	n.a.
14	74, m	HCC	cirrhosis	IIIB	151	8	A5	100	3.5, 3.9	abd. pain	A6 ^A^	PR	13.4	-	13.4	n.a.	13.4	17.2	pneumonia
15	65, m	HCC	NASH	II	60	5	A5	62	4.4	none	A5	PR	24.0	3.0	26.5	n.a.	26.5	29.0	-
17	82, m	HCC	cirrhosis	IB	323	32	A5	49	4.0	none	A5	SD	25.1	25.1	7.4	4.9	25.1	28.0	-
18	68, m	HCC	hemochrm.	IIIA	160	10	A5	89	3.9, 2.4	none	A6 ^A^	LTx	LTx	LTx	24.3	2.6	24.3	26.0	-
20	76, m	HCC	cirrhosis	IB	19	3	B7 ^A^	30	2.5	abd. pain	C10 ^A^	PD *	3.4	3.4	3.4	n.a.	3.4	21.4	HRS
4	58, m	mCRC	none	IVB	127	9	A6	100	3.6, 1.8	abd. pain	A6 ^A^	PD	2.6	-	2.6	1.5	7.2	23.8	prM, LF
5	62, f	mCRC	NAFLD	IVA	84	15	A5	25	2.2	none	A5	PR	Res	41.9	41.9	1.4	41.9	48.4	-
8	57, f	mCRC	none	IVA	571	25	A5	100	4.9, 4.2	none	A5	CR	11.8	-	3.2	0	19.6	35.5	n.a.
19	58, m	mCRC	none	IVA	291	22	A5	65	5.3	none	A6 ^A^	PD	1.5	1.5	13.7	0.8	13.9	28.8	LF
7	75, m	ICC	fibrosis	IIIB	240	25	A6	44	3.8	none	B7 ^A^	PR	5.2	2.9	2.9	n.a.	5.2	8.2	n.a.
16	71, f	HE	cirrhosis	IIIB	730	58	A6	52	5.0	none	B9 ^A^	n.a.	0.8	0.8	0.8	n.a.	0.8	22.3	HRS

* chronological numbering; TARE, transarterial radioembolization; HCC, hepatocellular carcinoma; ICC, intrahepatic cholangiocarcinoma; mCRC, metastatic colorectal cancer; HE, hemangioendothelioma; NAFLD, non-alcoholic fatty liver disease; NASH, non-alcoholic steatohepatitis; CPS, Child-Pugh score (A, with ascites); PD, progressive disease (* inferred); SD, stable disease; PR, partial remission; CR, complete remission; LF, acute liver failure; CRS, cardio-renal syndrome; HRS, hepato-renal syndrome; prM, progression of malignancy; LTx, liver transplantation before hepatic progression; Res, resection; n.a., not.

**Table 2:** Progression-free survival (PFS) in HCC and mCRC patients.

PFS after First ^166^Ho-TARE (Months)	HCC (14 Patients)	mCRC (4 Patients)
hepatic, treated liver	* 14.9 ± 11.6 (10.3, 8.8–21.0)	* 5.3 ± 5.7 (2.6, 2.4–8.3)
hepatic, untreated liver	* 10.9 ± 8.3 (8.8, 6.6–15.2)	21.7 ± 28.6 (21.7, 6.8–36.7)
hepatic, whole liver	8.9 ± 7.2 (6.4, 5.1–12.6)	14.5 ± 18.9 (7.2, 4.6–24.4)
extrahepatic	19.9 ± 16.0 (17.9, 11.6–28.3)	15.4 ± 18.4 (8.5, 5.7–25.0)
whole body	10.6 ± 9.7 (7.3, 5.5–15.7)	12.3 ± 19.8 (2.9, 2.0–22.7)

Values are mean ± SD (median, 95% confidence interval), * patients with liver transplantation and surgery after downstaging not included.

## Data Availability

Data sharing is not applicable to this article.
